# The association between alcohol consumption and osteoarthritis: a meta-analysis and meta-regression of observational studies

**DOI:** 10.1007/s00296-021-04844-0

**Published:** 2021-03-20

**Authors:** Kendrick To, Christopher Mak, Chen Zhang, Yuhui Zhou, Stephanie Filbay, Wasim Khan

**Affiliations:** 1 Division of Trauma and Orthopaedic Surgery, Department of Surgery, University of Cambridge, Cambridge, CB2 0QQ UK; 2 School of Clinical Medicine, University of Cambridge, Cambridge, CB2 0QQ UK; 3 Department of Physiotherapy, Centre for Health Exercise and Sports Medicine, University of Melbourne, Melbourne, VIC 3010 Australia; 4 Nuffield Department of Orthopaedics, Rheumatology and Musculoskeletal Sciences, University of Oxford, Oxford, OX3 7LD UK

**Keywords:** Osteoarthritis, Epidemiology, Alcohol, Meta-analysis

## Abstract

There is conflicting evidence for the association between alcohol consumption and common joint conditions such as Osteoarthritis (OA), which affects millions of people. We sought to determine the true association between alcohol intake and OA. We conducted a PRISMA systematic review and meta-analysis of observational studies that reported associations between alcohol consumption and OA. Pooled estimates of association were represented through odds ratios (ORs). Publication bias was assessed with Funnel and Galbraith plots, and risk of bias was assessed with the Newcastle Ottawa Scale. We included 29 studies and 25,192 subjects with OA and reported an OR between any alcohol consumption and OA of 0.79 (0.68–0.93), suggesting a protective effect. OR of weekly or more frequent use was 0.79 (0.65–0.97). When grouped by covariates, alcohol consumption was negatively associated with radiographic (0.83, 0.70–0.98), hand (0.80, 0.66–0.95) and knee OA (0.85, 0.72–0.99), North American ethnicity and female gender. Subgroup analysis of unadjusted data resulted in an OR of 0.70 (0.55–0.89) but this disappeared upon analysis of studies with data adjusted for any covariate (0.93, 0.78–1.10). Whilst our pooled analysis suggest that weekly or more frequent alcohol consumption was negatively associated with OA, this was not observed when adjusted for confounding factors. Reasons for this include selection bias and lack of longitudinal exposure and adjustment for confounding variables. Therefore, this meta-analysis provides evidence to dispel notions that alcohol use may be protective against OA.

**Supplementary Information:**

The online version contains supplementary material available at 10.1007/s00296-021-04844-0.

## Introduction

Alcohol consumption is a well-known risk factor for various major health conditions such as cerebrovascular disease, cardiovascular disease, and cancer. Reportedly, nearly 3 million deaths worldwide can be attributed to alcohol consumption each year [1]. Yet, there is conflicting evidence in the literature for the association of alcohol consumption with health. Several studies suggest that light to moderate alcohol consumption may have a negative association with specific outcomes of cardiovascular [2] and cerebrovascular disease [3]. The evidence for the beneficial effects of alcohol remains controversial. More recent large-scale international studies report that risk of all-cause mortality rises with alcohol consumption [4], and also that there were no risk thresholds beyond which a reduction in consumption would cease to confer risk reduction [5].

This debate extends into musculoskeletal disorders. The evidence that links alcohol use and musculoskeletal pain is tenuous. While it seems that situational pain due to musculoskeletal disease can increase risk of alcohol abuse as a means of self-medication [6, 7], others cite disease symptoms as reasons for alcohol cessation [8]. While alcohol use may be associated with increased levels of disease-relevant inflammatory markers in rheumatoid arthritis [9], other studies have shown that intake is inversely associated with disease [10, 11] in a dose-dependent manner [12], and is associated with improved function [13].

There are studies that suggest alcohol consumption may be a protective factor in Osteoarthritis (OA) [14]. This, however, is not consistently reported among observational studies. Possible mechanisms for this putative relationship include the association of alcohol use with reduced weight gain over time [15], but even this is an inconsistent finding, with some studies suggesting the opposite is true [16]. Alcohol may also have a positive association with physical activity [17], which may promote joint overuse, and therefore, increase risk of OA [18]. While there may be an underlying biological effect, with some studies in mouse reporting that alcohol consumption can lead to OA changes [19], the true effect of alcohol in human OA remains speculative.

The purpose of this meta-analysis of observational studies is to delineate the combined result between studies of the association between alcohol consumption and OA, and to determine whether subgroup analysis by subject demographics and diagnostic criteria can offer explanation to previously reported results. To our knowledge, this is the first meta-analysis to provide a pooled estimate of the association between alcohol consumption and risk of OA. Given the increasing global burden of OA and alcohol use [20, 21], understanding their association will inform public health policy and clinical risk prediction models.

## Materials and methods

### Literature search and study criteria

This meta-analysis was conducted in line with a protocol that adhered to Meta-analysis Of Observational Studies in Epidemiology (MOOSE) recommendations [22]. A literature search was conducted by three reviewers independently using EMBASE, PubMed via MEDLINE and Scopus databases. The captured publication dates spanned from inception to May 2020. The following search terms: ((((((((alcohol) OR alcoholic beverages) OR ethanol) OR beer) OR wine) OR liquor) OR drinking)) AND Osteoarthritis; were entered for PubMed. The Boolean operators were then adapted for other databases as appropriate.

Inclusion criteria were applied to all studies by each reviewer and disagreements were resolved through discussion. Titles and abstracts were screened for potential eligibility by three reviewers, and those satisfying inclusion criteria or where further information was required to determine eligibility, progressed to full-text review. Any disagreements were resolved through discussion and consensus. Eligibility criteria applied to titles and abstract included: full-text availability in the English language; the investigation of alcohol consumption as a potential risk factor for OA (diagnosed through self-report, clinical or radiological criteria); and a cohort, case–control, cross-sectional or randomised control trial (RCT) design. Subjects of all ethnicity or geographical habitation were included.

The eligibility criteria applied at full-text screen for inclusion in our meta-analysis were as follows:

1. Raw data allowing calculation of the quantitative association (in terms of odds ratio) between alcohol consumption (exposure) and diagnosis of OA.
  or
  Reporting of either Relative Risk (RR), Odds Ratios (OR) or Hazard Ratio (HR) with confidence intervals.
2. The inclusion of a comparison/control group of participants who abstain from alcoholic beverages.
3. Reporting of participant gender and age.
4. Methodology detailing study design including subject recruitment.
5. Information detailing methodology of establishing diagnosis of OA.

**Fig. 1:**
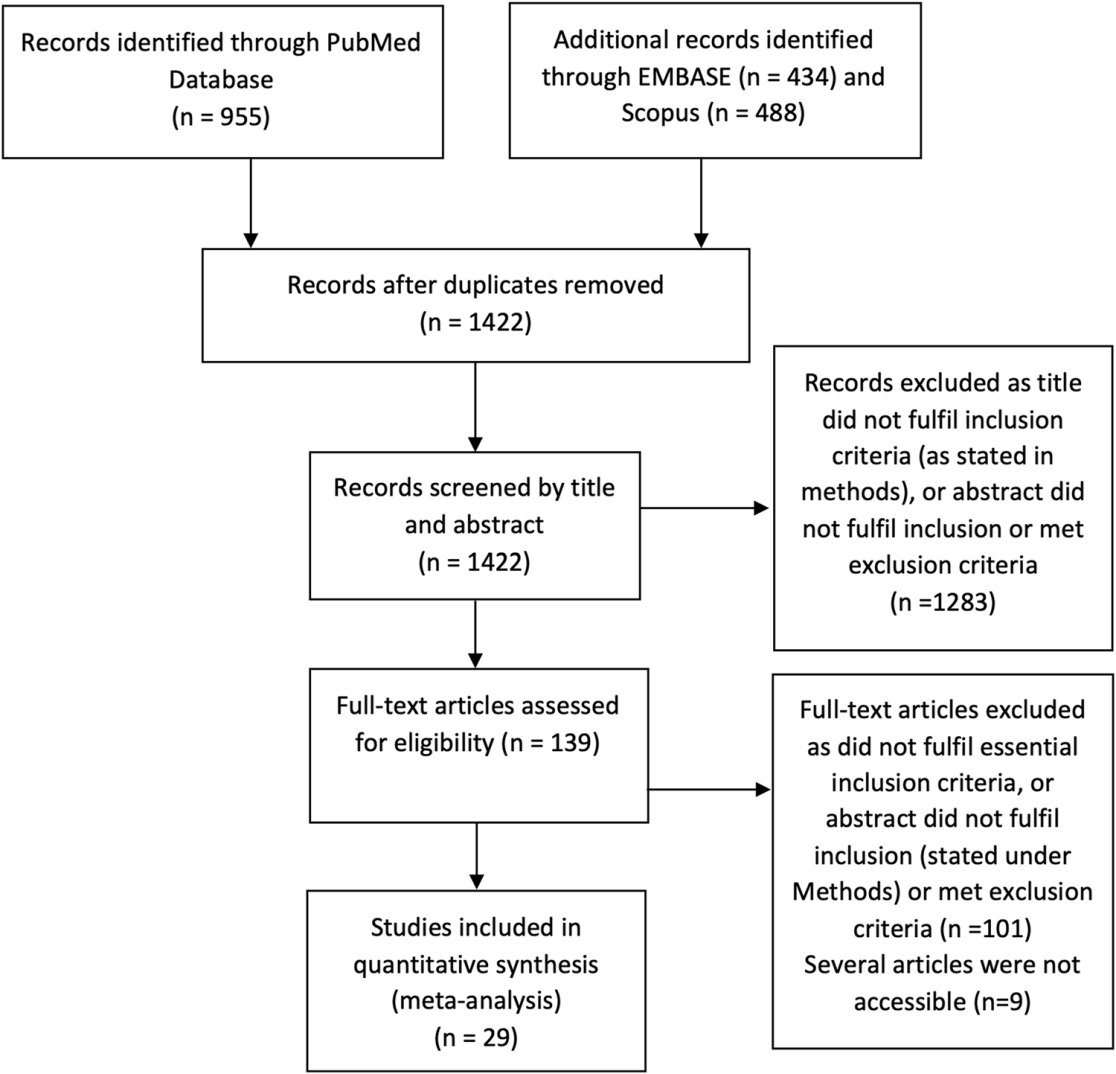
Flow diagram of literature search and study selection

Studies that defined OA through clinical endpoints such as having had joint replacement surgery were excluded. Systematic and literature reviews were excluded but to maximise capture, the bibliographies of these articles were scanned. The study selection process is illustrated in Fig. 1.

### Data extraction and risk of bias assessment

The population demographics of each study were extracted for analysis. Variables of interest included the quantity of alcohol consumed over a given time, and other cohort information as shown in Table 1. Cohort size, ethnicity, BMI, and gender distribution were extracted to facilitate sensitivity analysis based on subgroups. Funnel and Galbraith plots were generated to assess for publication bias. Asymmetry in the funnel plot and the gradient of the Galbraith plot were interpreted to determine the likely direction of bias in the association between alcohol consumption and OA. The risk of bias of each study was assessed using the Newcastle Ottawa Scale (NOS) [23].

**Table 1:** Baseline characteristics of included studies

Characteristics	Case–control	Cohort	Cross-sectional	Total
Number of studies	3	10	16	29
Patient sample size	2678	5892	16,622	25,192
Ethnicity (%)				
North American	0 (0)	4 (40.0)	5 (31.3)	9 (31.0)
European	3 (100)	2 (20.0)	4 (25.0)	9 (31.0)
East Asian	0 (0)	4 (40.0)	7 (43.8)	11 (37.9)
Mean age (range)	68.3 (64.0–70.0)	64.4 (41.8–70.5)	63.7 (38.0–75.6)	64.4 (38.0–75.6)
Mean BMI (range)	29.3 (26.4–30.3)	25.7 (23.1–27.8)	26.1 (24.1–29.6)	26.4 (23.1–30.3)
% Female (range)	53.7 (49.0–76.7)	57.0 (27.0–74.6)	65.7 (0.0–79.9)	62.4 (0.0–79.9)
% Smoker (range)	9.8 (9.0–10.0)	16.9 (7.1–40.6)	16.0 (9.3–63.8)	15.5 (7.1–63.8)
Diagnostic criteria (%)				
Clinical	0 (0)	1 (10.0)	1 (6.3)	2 (6.9)
Radiographic	1 (33.3)	6 (60.0)	7 (43.8)	14 (48.3)
Both	2 (66.7)	0 (0)	3 (18.8)	5 (17.2)
Self-reported	0 (0)	3 (30.0)	5 (31.3)	8 (27.6)
OA sites (%)*				
Hand	0 (0)	2 (20.0)	2 (12.5)	4 (13.3)
Knee	1 (25.0)	4 (40.0)	7 (43.8)	12 (40.0)
Hip	2 (50.0)	1 (10.0)	0 (0)	3 (10.0)
Spine	0 (0)	0 (0)	2 (12.5)	2 (6.7)
Multiple	1 (25.0)	2 (20.0)	2 (12.5)	5 (16.7)
Unspecified	0 (0)	1 (10.0)	3 (18.8)	4 (13.3)
Alcohol consumption pattern (%)^✝^				
Weekly or more frequent	3 (100)	5 (50.0)	7 (38.9)	15 (48.4)
Monthly	0 (0)	4 (40.0)	3 (16.7)	7 (22.6)
Undefined current drinkers	0 (0)	1 (10.0)	8 (44.4)	9 (29.0)

N.B. *hip and knee subgroups are stratified in Muthuri 2015 ^✝^weekly and monthly drinking subgroups are stratified in Bang 2018 and Magnusson 2017

### Data analysis

**Fig. 2:**
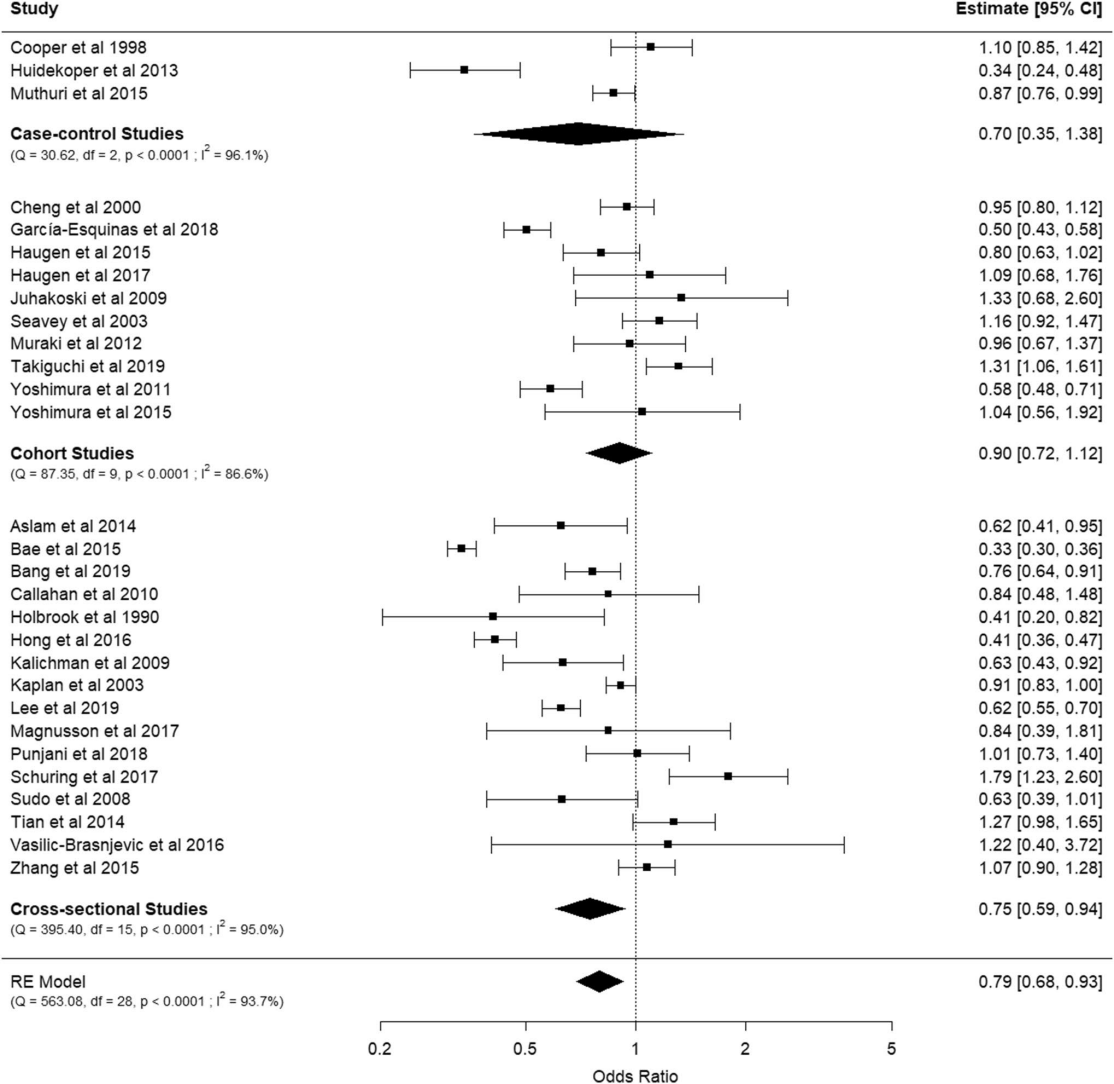
Summary forest plot of the association of alcohol consumption (any frequency) with osteoarthritis

**Fig. 3:**
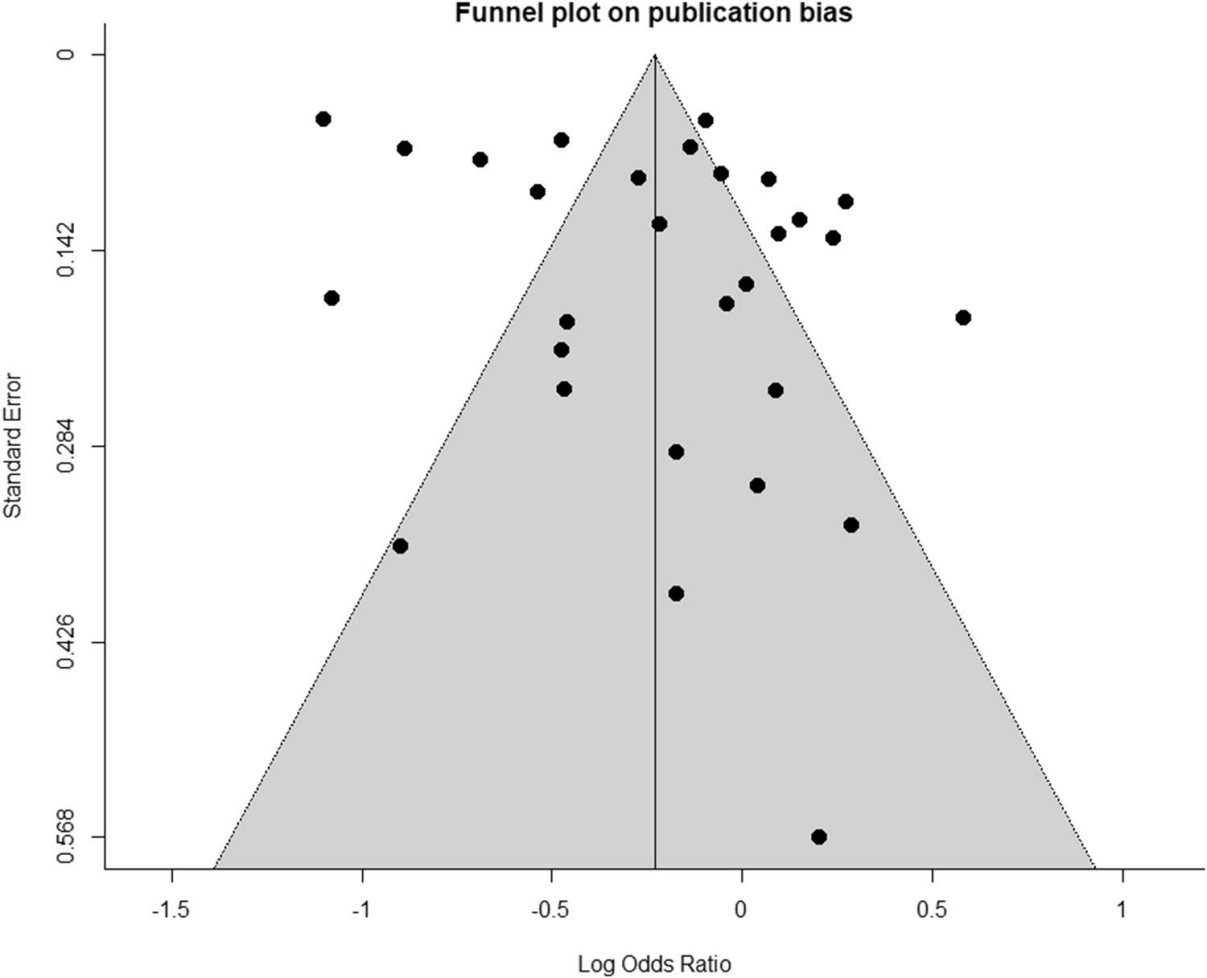
Funnel plot of data in summary analysis (CI: 95%). X-axis indicates effect size

ORs and confidence intervals (CI) were extracted for meta-analysis. RRs and HRs with CI or standard errors (SE) were converted to ORs through calculation using the metafor package on R software. Both confounder-adjusted and unadjusted OR were included for pooled meta-analysis. All data analyses were conducted using R version 4.0.0 software. A meta-analysis of all studies was performed to generate a summary forest plot (Fig. 2), stratifying studies by design. A funnel plot under a fixed-effects model was created to demonstrate the effect of SE and variance of individual studies (Fig. 3). This was visually inspected for asymmetry and evidence of publication bias.

**Fig. 4:**
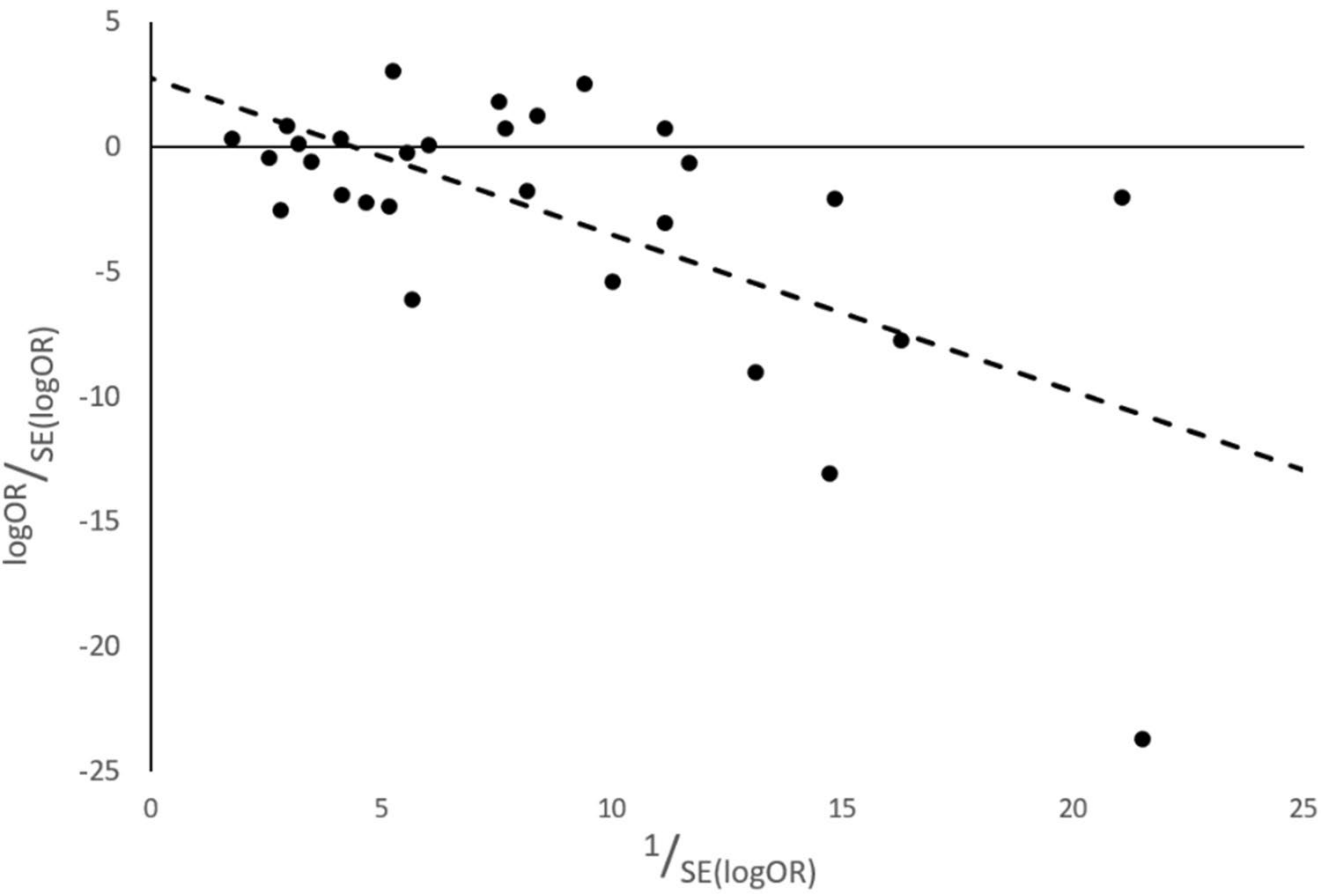
Galbraith plot for Egger’s test: *p* = 0.0003, *R*^2^ = 0.3889, Bias from Egger’s test: *α* = 2.7496, 95% CI − 0.3321 to 5.831

Heterogeneity was assessed by the *Q* test and *I*^2^ statistic performed on *R* 4.0.0 using the metafor package and RevMan 5.3 software, respectively. To further determine evidence for publication bias, a Galbraith plot for Egger’s test was generated (Fig. 4) and the *y*-intercept was reported.

**Fig. 5:**
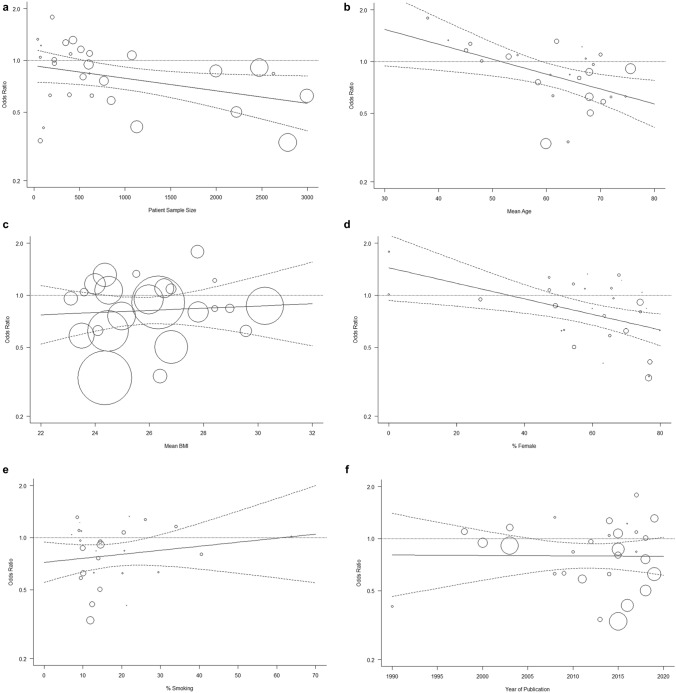
Bubble plots of subgroup analyses. **a** Patient sample size, **b** mean age, **c** mean BMI, **d** % female, **e** % smoking, and **f** year of publication N.B. circles representing patient sample size have been magnified by ×100 for visualisation purposes

Subgroup meta-analyses and data interpretation were performed according to the categories of study design, cohort size and various subject characteristics as grouped in Table 1. The categories of exposure in terms of level of alcohol consumption and outcomes measured in terms of diagnostic criteria used, and site of OA reported were grouped for analysis. Nearly all analyses reported a significant degree of heterogeneity (*I*^2^ > 50%) and a random effects model was applied for these to account for variability in variance. A fixed-effects model was applied for subgroup analysis of covariates that reported a low degree of heterogeneity (*I*^2^ < 25%). Likewise, all meta-regression analyses were performed under a restricted maximum likelihood (REML) approach. Meta-regression plots with ORs as the dependent variable were generated to illustrate the results of the analysed covariates graphically (Fig. 5). Only six covariates were analysed to avoid multiplicity. *R*^2^ statistic was used to determine the degree of fit, and the Tau statistic was used to measure heterogeneity.

## Results

### Study inclusion and characteristics

Our literature search captured a total of 1877 articles. After duplicates were removed, 1422 studies remained (Fig. 1). Following an initial title screen, 139 studies were assessed in full-text, and 9 articles were inaccessible. A total of 29 studies including 25,192 subjects with OA were deemed suitable for quantitative meta-analysis. All the included studies (Table 1) were observational in design; 16 were cross-sectional [24–39], 10 were cohort [40–49] and 3 were case–control studies [14, 50, 51]. There were no RCTs or prospective interventional studies yielded from our search. Eleven studies had cohorts with East Asian ethnicity, 9 studies had European cohorts and the remaining 9 had North American cohorts. The mean age of participants across all study designs were over 60 years, with an overall average of 64.4 (38.0–75.6) years. The average mean BMI was found to be 26.4 (23.1–30.3), the mean percentage of females included in the studies was 62.4 (0–79.9)% and an average mean of 15.5 (7.1–63.8)% were smokers.

Just under half of the studies (*n* = 14) determined OA by radiographic diagnosis, with Kellgren–Lawrence (KL) grade 2 or greater as the diagnostic threshold (Supplementary Table 1). Eight studies evaluated self-reported OA, seven of which reported a previous clinician diagnosis of OA [25, 27, 34, 37, 40, 41, 45], but did not report the criteria used by clinicians to make a diagnosis [26]. Two studies used clinical symptoms as a basis for diagnosis, one of which employed the American College of Rheumatology (ACR) criteria [51], and the other employed the European League Against Rheumatism (EULAR) criteria [30]. Twelve studies assessed OA in the knee, four in the hand, three in the hip and two in the spine. Five studies had cohorts with OA in various joints and four studies did not specify the joint site. Alcohol consumption patterns were stratified as weekly or more frequently, monthly, and as undefined current drinkers. Most studies specified drinkers that consumed alcohol weekly or more frequently and did not further define the upper limit of consumption. The characteristics of each individual study are shown in supplementary Table 1.

### Primary analyses of alcohol consumption and osteoarthritis

The meta-analysis of all 29 included studies reported an overall reduced odds of OA in people who consumed alcohol, compared to those who did not (OR, 95% CI: 0.79, 0.68–0.93), suggesting that alcohol has a protective effect (Fig. 2). Out of 29 studies, only 2 studies [27, 47] reported a statistically significant positive point estimate between alcohol use and OA, one study categorised alcohol use as > 1 g weekly [47], and the other employed the Alcohol Use Disorders Identification Test (AUDIT)-C score [27]. Conversely, ten studies [24, 25, 32, 34–36, 38, 41, 48, 51] found a statistically significant negative association.

When grouped by study design, the case–control studies reported a non-significant point estimate between alcohol use and OA. Within this group, only one study in which hand and foot OA were diagnosed through the ACR criteria, and alcohol use was categorised as > 1 glass weekly [51], found a significant and large protective effect associated with alcohol use.

The analysis of cohort studies included ten studies, which did not show a statistically significant association with an OR of 0.90 (0.72–1.12). Within this group, two studies reported a statistically significant negative association [41, 48] and one study reported a positive association [47]. Of the two studies that showed a negative association, one classified alcohol use as > 5 g of ethanol per day in a European population [41], and the other use a classification of > 1 drink per month in a Japanese population [48]. The study that demonstrated a positive association classified alcohol use as > 1 g of ethanol per week in a Japanese population [47]. The remainder of studies did not show a significant association. Despite the appearance of a uniform distribution of study effects based on visual inspection of the forest plot, a significant degree of heterogeneity was present (*I*^2^ = 86.6%, *p* < 0.0001).

Sixteen cross-sectional designs were included in our meta-analysis. Within this subgroup, seven studies showed a statistically significant negative association between alcohol use and OA and one study showed a significant positive association. It is, therefore, not unexpected that a significant degree of heterogeneity was present (*I*^2^ = 95.0%). An overall odds ratio of 0.75 (0.59–0.94) was shown, suggesting that the negative association seen in the primary analysis of all 29 studies is likely attributed to data from cross-sectional studies.

### Analyses of subject characteristics as covariates

Ethnicity was examined as a covariate, with only North American subjects showing a negative association between alcohol use and OA (Table 2). When stratified for gender, analysis of studies (*n* = 6) that had majority male subjects showed no significant association. Conversely, a significant negative association (0.72, 0.59–0.88) was observed in the analysis of the 23 studies with majority female subjects. Regressing the odds ratios by the average BMI of subjects did not reveal any significant associations (Table 3).

**Table 2:** Subgroup meta-analyses of the association between alcohol consumption and osteoarthritis by study variables

	Number of studies	Number of subjects	Odds ratio (95% CI)	*I*^2^ for heterogeneity (%)	*p* value for heterogeneity
Patient sample size					
*N* < 500	13	2767	0.91 (0.69–1.20)	84	< 0.0001
*N* ≥ 500	16	22,425	0.73 (0.58–0.91)	97	< 0.0001
*N* ≥ 1000	8	17,294	0.64 (0.46–0.89)	98	< 0.0001
Ethnicity					
North American	9	8120	0.91 (0.85–0.98)	45	0.07
European	9	6221	0.82 (0.59–1.14)	91	< 0.0001
East Asian	11	10,851	0.74 (0.54–1.01)	97	< 0.0001
Mean BMI					
< 25	10	9888	0.78 (0.55–1.10)	97	< 0.0001
≥ 25	14	13,110	0.84 (0.70–1.02)	87	< 0.0001
Undefined	5	2194	0.68 (0.39–1.18)	94	< 0.0001
Predominant sex					
Male majority	6	4440	1.08 (0.91–1.27)	72	0.003
Female majority	23	20,752	0.72 (0.59–0.88)	95	< 0.0001
Diagnostic criteria					
Clinical	2	120	1.30 (0.73–2.31)	0	0.90
Radiographic	14	9782	0.83 (0.70–0.98)		< 0.0001
Both	5	6171	0.66 (0.41–1.08)	96	< 0.0001
Self-reported	8	9119	0.78 (0.51–1.17)	98	< 0.0001
OA sites					
Hand	4	1945	0.80 (0.66–0.95)	4	0.37
Knee	12	12,765	0.85 (0.72–0.99)	84	< 0.0001
Hip	3	1645	1.01 (0.84–1.21)	13	0.32
Spine	2	1477	0.72 (0.24–2.17)	98	< 0.0001
Multiple	5	1494	0.80 (0.49–1.30)	92	< 0.0001
Unspecified	4	5866	0.56 (0.36–0.85)	95	< 0.0001
Alcohol consumption pattern					
Weekly or more frequent	15	14,292	0.79 (0.65–0.97)	94	< 0.0001
Monthly	7	5234	0.73 (0.46–1.15)	96	< 0.0001
Undefined current drinkers	9	5666	0.88 (0.70–1.12)	71	0.0006
Confounding					
Unadjusted	15	15,903	0.70 (0.55–0.89)	96	< 0.0001
Adjusted for any	14	9289	0.93 (0.78–1.10)	77	< 0.0001
Adjusted for BMI	9	7468	0.88 (0.70–1.11)	85	< 0.0001
Adjusted for age	13	8678	0.92 (0.76–1.12)	80	< 0.0001
Adjusted for gender	10	5232	0.90 (0.71–1.12)	80	< 0.0001
Adjusted for smoking	8	5852	0.84 (0.62–1.13)	85	< 0.0001

**Table 3:** Meta-regression analyses of select covariates

	Α	Β	SE	Τ	*I*^2^ (%)	*R*^2^ (%)	*Z *score	*p* value
Sample size	− 0.071	− 0.00017	0.0001	0.372	92.47	13.21	− 2.0003	0.0455
Mean age	1.031	− 0.01998	0.0076	0.345	91.00	20.40	− 2.6376	0.0084
Mean BMI	− 0.582	0.01464	0.0448	0.397	93.62	< 0.01	0.3268	0.7438
% Female	0.366	− 0.01034	0.0036	0.346	91.57	24.83	− 2.8535	0.0043
% Smoker	− 0.329	0.00536	0.0062	0.355	92.56	0.29	0.8640	0.3876
Year of publication	0.923	− 0.00057	0.0123	0.407	93.56	< 0.01	0.0465	0.9629

### Osteoarthritis diagnosis and site

Only OA diagnosed radiographically showed a significantly negative association (0.83, 0.70–0.98). This group also had the greatest number of studies (*n* = 14) and included 9782 subjects. When analysing studies that combined both clinical and radiographic evidence as diagnostic criteria, no significant association was found (0.66, 0.41–1.08).

Studies of knee OA predominated in our study, with 12,765 subjects across 12 studies. Meta-analyses grouped by this covariate, along with hand OA (0.80, 0.66–0.95) and site-unspecified OA (0.56, 0.36–0.85) found significant negative associations. There were no significant associations reported in the pooled analysis of hip OA, spine OA or multi-site OA subgroups.

### Analyses of alcohol consumption pattern and risk of osteoarthritis

Owing to the differences in study design and heterogeneity among studies, the amount of alcohol use in the exposed subjects was further stratified into three categories: weekly or more frequent (*n* = 15 studies), monthly (*n* = 7), and undefined current drinkers (*n* = 9). Only the group that included weekly or more frequent drinkers, which encompassed 14,292 subjects (Table 2), found a statistically significant point estimate of 0.79 (0.65–0.97) and with an *I*^2^ value of 94% (*p* < 0.0001). The other two categories did not identify any significant association. It was not possible to determine the upper limit of alcohol consumed in terms of ethanol units per time, as this was not detailed within nearly all of the studies.

### Meta-analyses of confounding factors

We found that a large study sample size (*n* > 500 and *n* > 1000) was related to significantly negative associations between alcohol use and OA. Whereas stratifying for studies with cohort sizes less than 500 (*n* = 13) did not show a significant association. The negative association found in the primary analyses was present on subgroup analysis of studies that did not adjust for covariates (0.70, 0.55–0.89) but did not remain after adjustment for any confounders (BMI, age, gender or smoking).

### Meta-regression analyses

Regression analyses showed that mean age, % female within the study and sample size were covariates that demonstrated a significant association with the ORs of association between alcohol use and OA reported by individual studies (Table 3). A negative association was seen with increasing age (Fig. 5b), increasing % female (Fig. 5d) within the cohort samples and with increasing sample size (Fig. 5a), this was consistent with the subgroup meta-analyses performed on these covariates.

### Risk of bias

We determined the number of studies with adjustments for confounding factors in their individual analyses and found that data extracted from 15 studies were unadjusted. Fourteen studies adjusted for either BMI, age, gender or smoking as confounding variables. A funnel plot of the summary meta-analysis (Fig. 3) demonstrated relative symmetry in study distribution through visual inspection, but when displayed as a Galbraith plot for Egger’s test (Fig. 4), the y-intercept value and negative gradient suggested a bias towards studies reporting negative associations. Risk of bias assessment was carried out using the Newcastle Ottawa Scale (NOS) [23]. The mean scores within all three study designs exceeded 70% of the maximum score (Supplementary Tables 2, 3, 4) suggesting overall good study quality [23]. Five studies were rated with a total score of five or less, and these were attributed to poor adherence to the recommendations in the selection section of the NOS.

## Discussion

Although our primary analysis revealed a statistically significant negative association between alcohol use and OA, subgroup analyses revealed that this is likely to be an unreliable conclusion. When studies were grouped by adjustment, an analysis of the 16 studies that contributed unadjusted data revealed an OR of 0.70 (0.55–0.89). This was a more negative association than that of the primary analysis which reported an OR of 0.79 (0.68–0.93). When grouped by any adjustment, this negative association was no longer observed.

These results suggest that any negative association seen in the summary analysis was probably due to a lack of confounder adjustment in individual studies. We found that there were several covariates which could explain this. When exploring cohort characteristics, a negative association was seen in weekly or more frequent drinkers, radiographically diagnosed OA, hand OA, knee OA, and site-unspecified OA. It was also seen in North American cohorts and cohorts with female-predominant subjects. When grouped by cohort size, studies that contributed over 500 or over 1000 subjects appeared to show a significant negative association. Consistent with results of the meta-analyses, our meta-regression analyses found negative associations with age, female gender, and study sample size.

It also appeared that study design played a significant role. The subgroup analysis for cross-sectional studies supported the negative association observed in the primary analysis, whereas no association was seen when stratified by other study designs.

### Strengths of the study

To our knowledge, this meta-analysis is the first to provide a pooled estimate of the association between alcohol consumption and risk of OA. Other meta-analyses have employed similar methodology to assess the association between alcohol consumption and rheumatoid arthritis [12], and between smoking and OA [52], but not between alcohol consumption and OA. No eligible RCTs were identified, and analyses of published observational studies included both cross-sectional and prospective studies. This allowed bias relating to study design to be determined [53]. We further conducted meta-regression analyses for several study characteristics. Inclusion of referent groups from multiple study designs and geography may allow for the effects of inter-study uncontrolled confounding factors such as population health profiles that vary according to geography, to be mitigated. Indeed, alcohol consumption varies significantly between countries [54], but may also vary within a population [55]. We also included studies that provided both adjusted and unadjusted data, and so allowed for the influence of adjustment to be observed in the subgroup analyses. For grouped analyses, a threshold of *I*^2^ = 25% or lower was adopted for the application of a fixed-effects model in meta-analyses. Use of higher thresholds may erroneously report statistically significant associations [56]. To confirm results of the *I*^2^ test, a *Q*-statistic was calculated for our primary analysis.

The frequency of alcohol consumption was categorised to determine whether any associations observed may be dose-related and specific to a pattern of use. This is important as referent cohorts are likely biased towards common habits of alcohol consumption, which tends to be more frequent than monthly use [57].

In addition to this, we stratified OA by method of diagnosis. This may be informative as the use of alcohol could potentially interfere with clinical symptoms of OA and thereby obscure any association seen with clinical OA. On the contrary, radiographic assessment allows the degree of structural OA changes to be quantified. Interestingly, when grouped into such classifications, our analysis showed that only radiographic OA had a negative association with alcohol consumption. A potential explanation for this could be that individuals with symptomatic OA are more likely to consume alcohol as a means of analgesia [7].

### Interpretation of findings and implications for policy

In our meta-analysis, we show that the reported negative associations between alcohol use and OA are likely untrue. In support of this, analysis of studies adjusted for confounders eradicated any significant associations and suggested that alcohol had no significant association with OA. We further attempted to reduce heterogeneity by assessing adjustment by different covariates but were not successful. Most of the heterogeneity seem to arise from the OA joint of interest, as hand OA and hip OA groups both demonstrated low heterogeneity alone and did not show a significant association.

The basis for how alcohol affects OA can be primary or secondary. There is no clear consensus on what the primary biological effects may be. It is known that alcohol use can increase uric acid levels and thus increase the risk of inflammatory joint conditions such as gout [58]. In OA, synovial fluid uric acid levels appear to correlate with pro-inflammatory cytokines and radiographic OA severity [59], suggesting a possible causal link. In vivo studies support this, as alcohol consumption in mice seem to drive structural OA changes over time [19]. Conversely, resveratrol which is present in wine has been shown to exhibit chondroprotective effects in vitro [60, 61] and so may explain the findings in Muthuri’s study [14]. This, rather than alcohol itself is a possible explanation for the suggested protective effects of alcoholic beverages. Alcohol can also have secondary effects that contribute to OA risk. For example, alcohol use is associated with obesity [62, 63], and this may inadvertently promote joint loading and increased risk of OA in healthy individuals [64]. Conversely, appropriate joint-use and loading is recommended in individuals with existing OA to attenuate symptoms [65] without causing structural disease progression [66]. Physical activity, among other interventions, aimed at certain muscle groups such as femoral strengthening exercise for knee OA, may be beneficial for OA in particular joints [67]. The potential effects of heavy alcohol consumption on reduced muscle strength [68] could also reduce the potential benefits of exercise on early OA. Existing literature does not agree on the relationship between alcohol use and OA, with some reporting no association [69] and others reporting a positive association [17]. The contention extends to other aspects of the musculoskeletal system, with studies reporting beneficial effects of light to moderate alcohol consumption on bone mineral density and detrimental effects of heavy alcohol consumption [70]. Other literature suggests that like heavy drinkers, non-drinkers are also at greater risk of osteoporosis compared with light drinkers [71].

### Limitations of the study

The majority of the meta-analyses displayed a substantial degree of heterogeneity, and so interpretations should be made with caution [53]. As only observational studies were identified, there was inherently low internal validity. In addition, our primary analysis was biased towards cross-sectional and cohort studies which comprised the bulk of included studies [72]. Whilst publication bias was not obvious on a funnel plot, Egger’s test was conducted as an adjunct to improve the reliability of bias assessment [73]. This revealed a bias towards negative associations and was consistent with the point estimate observed in the primary analysis. Whilst we analysed studies grouped by absence or presence of confounder adjustment, the adjustment methodology varied significantly between individual studies. This heterogeneity can have greater impact on pooled estimates that report a weak association [74], which consistent with the findings in this meta-analysis. Objective stratification of studies by the comprehensiveness of the adjustment methodology may, therefore, be beneficial [75], but this was not done, due to the small number of studies that performed covariate adjustment, and to avoid multiplicity. The quality assessment carried out in accordance with the NOS suggested that the majority of studies were subject to selection bias (Supplementary Tables 2, 3 and 4). To overcome limitations in previous studies, future research should aim to account for non-responders in all groups and employ previously validated tools to assess OA.

Estimates of alcohol use were largely subjective and self-reported. Therefore, alcohol consumption is likely to be underestimated, especially in heavy use [76]. This probably exaggerates the effect of any observed association. While our analysis did stratify alcohol consumption, the upper limits of alcohol consumption were poorly defined in the studies and a dose–response relationship was not assessed beyond weekly or more frequent use. Some meta-analyses have found that alcohol consumption beyond a certain amount confers risk, whereas moderate quantities may be protective for particular diseases [2]. As the amount of alcohol consumed weekly can vary significantly, we were unable to evaluate such relationships in our study. The predominance of cross-sectional studies in our meta-analyses is another source of inaccuracy in estimating alcohol use, which is known to vary seasonally [77]. In addition to this, there is literature to suggest that symptoms of musculoskeletal disease may lead to greater alcohol consumption as a means of analgesia [7]. Chronic musculoskeletal pain can be comorbid to depressive mood disorder [78] which can also be associated with heavier use [79]. These influences are likely to generate bias towards a positive association between alcohol use and OA. As none of the included studies conducted quantitative adjustments for mental health status and pain as a covariate, it is an important consideration for future research. Indeed, the study in this meta-analysis that reported the greatest positive association derived their results from a cohort of subjects exclusively with adverse levels of alcohol use [27]. Gender also plays a role in alcohol consumption, with significantly less heavy-users in female cohorts [47]. Female gender is also known to have a positive association with OA [80], and so an analysis with a greater proportion of female subjects may polarise towards a negative association. An important limitation in this study pertains to the differing bioactive contents between alcoholic beverages. One study [14] assessed both beer and wine consumption and reported that the former was a risk factor whereas the latter was protective for OA. Inferences from a pooled analysis could, therefore, be susceptible to bias by confounders such as socio-economic status which can influence choice of alcohol beverage [81]. Regardless, as it is known that particular constituents in wine can influence cardiovascular disease risk, their possible effects in musculoskeletal disease cannot be disregarded.

It is difficult to determine the most suitable inclusion criteria for the presence of OA. Structural changes can be reliably determined radiographically [82], whereas the clinical relevance of a meta-analysis may be enhanced if focused on clinically diagnosed disease. Self-reported OA is unreliable as subjective recollection of a previous clinical diagnosis, which most of the studies in this meta-analysis relied on, can potentially be biased, and self-reported symptoms may not be robust indicators of disease, and symptoms may even be absent in the presence of structural disease [83]. These factors contributed to the heterogeneity of our analysis, with there being the greatest heterogeneity within the self-reported OA group, and least in the clinical OA group. Future studies should aim to adopt case–control designs to delineate dose–response relationships between alcohol use and joint-specific OA diagnosed through validated clinical and radiographic criteria. Covariate adjustment should be based on possible confounders to joint disease and symptoms such as diet and degree of activity level as well as baseline health characteristics such as age, gender and BMI. As the inter-ethnic effects of alcohol on health can be discordant [84], partly due to underlying variations in genetic polymorphisms that affect alcohol metabolism [85], we recommend analysing ethnicity as a factor in future studies.

## Conclusion

Our meta-analysis shows that previous reports of a protective effect of alcohol consumption on risk of osteoarthritis are likely to be inaccurate. The suggested protective effect is likely to have been observed due to lack of confounder adjustment. Alcohol use is a public health issue and with mounting global disease burden attributable to alcohol misuse [54] it is increasingly important that recommendations for safe consumption are supported by robust research. Failure in this avenue can result in harm at a population level [86]. It is probable that most individuals will be deterred from alcohol use in attempt to alleviate joint disease [8]. In contrast, certain individuals who suffer from joint pain may increase alcohol use for temporary symptom relief [7]. For this group, it is important that advice on consumption is not based on misinterpretations of falsely reported negative associations between alcohol and OA. This meta-analysis provides evidence to dispel notions that alcohol use may protect against OA.

## Supplementary Information

Below is the link to the electronic supplementary material.

Supplementary file1 (DOCX 31 KB)
