# Supplementary material for: The association between alcohol consumption and osteoarthritis: a meta-analysis and meta-regression of observational studies
Source: Rheumatol Int. 2021 Mar 20;41(9):1577–91. doi: 10.1007/s00296-021-04844-0 (PMC8316228; doi:10.1007/s00296-021-04844-0)
Supplement: Supplementary file 1 — Supplementary file1 (DOCX 31 KB) [file 296_2021_4844_MOESM1_ESM.docx]

| **Study** | **Study Design** | **Sample size** | **Ethnicity** | **Mean age (years)** | **Mean BMI** | **% Female** | **% Smoker** | **OA site** | **Diagnostic criteria** | | **Alcohol consumption pattern (frequency and method of measurement)** | | **Adjustment for covariates** |
| --- | --- | --- | --- | --- | --- | --- | --- | --- | --- | --- | --- | --- | --- |
| Aslam et al 2014 | Cross-sectional | 638 | US | 71.9 | 29.6 | 50.9 | 20.2 | Unspecified | Radiographic | Kellgren Lawrence | Current | yes/no | unadjusted |
| Bae et al 2015 | Cross-sectional | 2786 | Korean | 59.9 | 24.4 | 76.6 | 11.9 | Unspecified | Self-reported | Clinical symptoms | Monthly | ≥ once a month | unadjusted |
| Bang et al 2019 | Cross-sectional | 770 | Korean | 58.4 | 25.0 | 63.5 | 14.0 | Knee | Radiographic | Kellgren Lawrence | Weekly  Monthly | >1 glass/month | unadjusted |
| Callahan et al 2010 | Cross-sectional | 2627 | US | 60.7 | 29.0 | 59.9 | 20.8 | Knee | Radiographic and clinical | Kellgren Lawrence and Clinical symptoms | Current | yes/no | Body Mass Index (BMI), age, ethnicity, smoking injury |
| Cheng et al 2000 | Cohort | 601 | US | NA | 26.0 | 27.0 | 14.6 | Knee and hip | Self-reported | Clinical symptoms | Weekly | >0.01g/week | unadjusted |
| Cooper et al 1998 | Case-control | 611 | UK | 70.0 | 26.6 | 65.6 | 9.0 | Hip | Radiographic | Kellgren Lawrence | Weekly | ≥1 units/week | BMI, injury, Heberden nodes |
| García-Esquinas et al 2018 | Cohort | 2221 | UK and Spain | 68.1 | 26.8 | 54.6 | 14.4 | Unspecified | Self-reported | Clinical symptoms | Weekly | ≥5g/day | unadjusted |
| Haugen et al 2015 | Cohort | 540 | US | 66.1 | 27.8 | 74.2 | 40.6 | Hand | Radiographic | Kellgren Lawrence | Current | yes/no | unadjusted |
| Haugen et al 2017 | Cohort | 403 | US | 54.7 | 26.8 | 57.8 | 9.7 | Hand | Radiographic | Kellgren Lawrence | Weekly | ≥1 drinks/week | age, sex, education |
| Holbrook et al 1990 | Cross-sectional | 106 | US | NA | NA | 63.1 | 21.3 | Hand, knee, hip and spine | Self-reported | Clinical symptoms | Current | yes/no | unadjusted |
| Hong et al 2016 | Cross-sectional | 1129 | Korean | NA | NA | 77.0 | 12.4 | Spine | Radiographic and clinical | Not specified | Weekly | heavy drinking | unadjusted |
| Huidekoper et al 2013 | Case-control | 73 | Dutch | 64.0 | 26.4 | 76.7 | NA | Hand and feet | Radiographic and clinical | American College of Rheumatology (ACR) Criteria of Clinical symptoms | Weekly | >1 glass/week | BMI, age, sex, smoking |
| Juhakoski et al 2009 | Cohort | 41 | Finnish | 41.8 | 25.5 | 58.5 | 22.0 | Hip | Clinical | Clinical symptoms | Weekly | >1g/week | BMI, age, sex, smoking, injury |
| Kalichman et al 2009 | Cross-sectional | 390 | Russian | 61.2 | NA | 51.6 | 29.5 | Hand | Radiographic | Kellgren Lawrence | Weekly | ml/week | BMI, age, smoking |
| Kaplan et al 2003 | Cross-sectional | 2470 | Canadian | 75.6 | 26.3 | 74.1 | 14.6 | Knee | Self-reported | Clinical symptoms | Weekly | >1 drinks/week | unadjusted |
| Lee et al 2019 | Cross-sectional | 2991 | Korean | 67.9 | 24.5 | 69.9 | 10.1 | Knee | Radiographic | Kellgren Lawrence | Weekly | ≥2 drinks/week | unadjusted |
| Magnusson et al 2017 | Cross-sectional | 612 | Norwegian | 64.3 | 28.4 | 76.0 | 13.4 | Hand | Radiographic | Kellgren Lawrence | Weekly  Monthly | yes/no  yes/no | age, sex, education |
| Muraki et al 2012 | Cohort | 227 | Japanese | 68.7 | 23.1 | 66.3 | 9.4 | Knee | Radiographic | Kellgren Lawrence | Monthly | yes/no | BMI, age, sex, smoking, injury |
| Muthuri et al 2015 | Case-control | 1994 | UK | 68.0 | 30.3 | 49.2 | 10.0 | Knee and hip | Radiographic and clinical | Kellgren Lawrence and clinical symptoms | Weekly | >1 drink/week | BMI, age, sex, smoking, injury, renal disease, occupational risks, physical activity, type of beverages |
| Punjani et al 2018 | Cross-sectional | 221 | Canadian | 48.0 | NA | 0.0 | 63.8 | Unspecified | Self-reported | Clinical symptoms | Current | Alcohol Use Disorders Identification Test (AUDIT)-C score ≥3 | age, sex, ethnicity, employment, geographic location, living conditions, education, income |
| Schuring et al 2017 | Cross-sectional | 200 | European | 38.0 | 27.8 | 0.0 | NA | Knee and spine | Self-reported | Clinical symptoms | Current | AUDIT-C score ≥3 | unadjusted |
| Seavey et al 2003 | Cohort | 514 | US | 45.1 | 24.0 | 54.4 | 34.0 | Hand and knee | Self-reported | Clinical symptoms | Monthly | >1 drink/month | age, sex, and ethnicity |
| Sudo et al 2008 | Cross-sectional | 179 | Japanese | 74.7 | 24.1 | 79.9 | 12.8 | Knee | Radiographic | Kellgren Lawrence | Current | yes/no | unadjusted |
| Takiguchi et al 2019 | Cohort | 429 | Japanese | 61.9 | 24.4 | 67.8 | 8.6 | Knee | Radiographic | Kellgren Lawrence | Weekly | >1g ethanol/week | Male: BMI, age, smoking, green tea consumption  Female: BMI, age, household income |
| Tian et al 2014 | Cross-sectional | 348 | Chinese | 45.9 | NA | 47.3 | 26.2 | Spine | Radiographic and clinical | Kellgren Lawrence and clinical | Weekly | ≥1000mL beer or 100mL liquor/week | unadjusted |
| Vasilic-Brasnjevic et al 2016 | Cross-sectional | 79 | Serbian | 66.6 | 28.4 | 68.7 | 9.3 | Knee | Clinical | European League Against Rheumatism (EULAR) criteria | Current | yes/no | unadjusted |
| Yoshimura et al 2011 | Cohort | 845 | Japanese | 70.5 | 23.5 | 65.1 | 9.5 | Knee | Radiographic | Kellgren Lawrence | Monthly | >1 drink/month | unadjusted |
| Yoshimura et al 2015 | Cohort | 71 | Japanese | 67.3 | 23.6 | 74.6 | 7.1 | Knee | Radiographic | Kellgren Lawrence | Monthly | ≥ once a month | age, sex, smoking, geographical location |
| Zhang et al 2015 | Cross-sectional | 1076 | Chinese | 53.0 | 24.5 | 47.4 | 20.6 | Knee | Radiographic | Kellgren Lawrence | Current | yes/no | BMI, age, sex, physical activity, education, calorie intake, betel quid chewing |

Supplementary Table 1. Characteristics of individual studies.

| **Study** | **Selection** | **Comparability** | **Outcome** | **Total Score (max = 9)** |
| --- | --- | --- | --- | --- |
| Cheng et al 2000 | 3 | 2 | 2 | 7 |
| Garcia-Esquinas et al 2018 | 2 | 2 | 1 | 5 |
| Haugen et al 2017 | 4 | 2 | 3 | 9 |
| Juhakoski et al 2009 | 4 | 2 | 2 | 8 |
| Muraki et al 2012 | 4 | 2 | 2 | 8 |
| Takiguchi et al 2019 | 4 | 2 | 3 | 9 |
| Yoshimura et al 2011 | 3 | 2 | 3 | 8 |
| Yoshimura et al 2015 | 3 | 2 | 3 | 8 |
| Mean Score | 7.75 |  |  |  |

Supplementary Table 2. Quality assessment of individual cohort studies based on the Newcastle Ottawa Scale. Selection (maximum score of 4), Comparability (maximum score of 2), Outcome (maximum score of 3).

| **Study** | **Selection** | **Comparability** | **Outcome** | **Total Score (max = 10)** |
| --- | --- | --- | --- | --- |
| Aslam et al 2014 | 5 | 2 | 3 | 10 |
| Bae et al 2015 | 4 | 2 | 2 | 8 |
| Bang et al 2019 | 4 | 2 | 3 | 9 |
| Callahan et al 2010 | 4 | 2 | 3 | 9 |
| Holbrook et al 1990 | 1 | 1 | 2 | 4 |
| Hong et al 2016 | 4 | 2 | 3 | 9 |
| Kalichman et al 2009 | 4 | 2 | 3 | 9 |
| Kaplan et al 2003 | 2 | 2 | 2 | 6 |
| Lee et al 2019 | 5 | 2 | 2 | 9 |
| Magnusson 2017 | 3 | 2 | 3 | 8 |
| Punjani et al 2018 | 1 | 2 | 2 | 5 |
| Schuring et al 2017 | 2 | 1 | 2 | 5 |
| Sudo et al 2008 | 3 | 0 | 2 | 5 |
| Tian et al 2014 | 4 | 2 | 2 | 8 |
| Vasilic-Brasnjevic et al 2016 | 5 | 2 | 2 | 9 |
| Zhang et al 2015 | 5 | 2 | 2 | 9 |
| Mean Score | 7.63 |  |  |  |

Supplementary Table 3. Quality assessment of individual cross-sectional studies based on the Newcastle Ottawa Scale. Selection (maximum score of 5), Comparability (maximum score of 2), Outcome (maximum score of 3).

| **Study** | **Selection** | **Comparability** | **Exposure** | **Total Score (max = 9)** |
| --- | --- | --- | --- | --- |
| Cooper et al 1998 | 4 | 2 | 2 | 8 |
| Huidekoper et al 2013 | 4 | 1 | 1 | 6 |
| Muthuri et al 2015 | 4 | 2 | 2 | 7 |
| Mean Score | 7 |  |  |  |

Supplementary Table 4. Quality assessment of individual case-control studies based on the Newcastle Ottawa Scale. Selection (maximum score of 4), Comparability (maximum score of 2), Exposure (maximum score of 3).
